# Supplementary material for: What Is a 2021 Reference Body?
Source: Nutrients. 2022 Apr 6;14(7):1526. doi: 10.3390/nu14071526 (PMC9003358; doi:10.3390/nu14071526)
Supplement: Supplementary file 1 [file nutrients-14-01526-s001.zip › nutrients-1640856-supplementary.pdf]

Supplement: Table S1. Characteristics of the adults used in developing the manifold regression equations.

	Male			Female	
	<40	≥40	Total	<40	≥40
**N (%)**	115 (44.6)	143 (55.4)	258 (45.3)	121 (38.8)	191 (61.2)
**Ethnicity**					
**Asian**	30 (26.1%)	29 (20.3%)	59 (22.9%)	26 (21.5%)	42 (22.0%)
**Black**	20 (17.4%)	35 (24.5%)	55 (21.3%)	22 (18.2%)	45 (23.6%)
**Hispanic**	20 (17.4%)	10 (7.0%)	30 (11.6%)	23 (19.0%)	21 (11.0%)
**NHOPI**	11 (9.6%)	4 (2.8%)	15 (5.8%)	13 (10.7%)	11 (5.8%)
**White**	34 (29.6%)	65 (45.5%)	99 (38.4%)	37 (30.6%)	72 (37.7%)
**Age (years)**					
**Mean (SD)**	28.7 (6.20)	55.9 (10.0)	43.8 (16.0)	28.7 (5.97)	56.6 (9.02)
**Median [range]**	29.0 [18,39]	57.0 [40,79]	42.5 [18.0-79.0]	29.0 [18,39]	58.0 [40,75]
**Height (cm)**					
**Mean (SD)**	176 (7.74)	176 (7.49)	176 (7.59)	163 (7.22)	162 (6.80)
**Median [range]**	176 [155,202]	176 [151,190]	176 [151-202]	163 [146,181]	162 [144,181]
**Weight (kg)**					
**Mean (SD)**	85.1 (19.3)	88.8 (21.1)	87.2 (20.4)	73.4 (22.1)	70.9 (19.3)
**Median [range]**	84.9 [41.5,174]	82.5 [40.6,149]	83.8 [40.6-174]	68.6 [38.6,153]	68.6 [35.4,146]
**BMI (kg/m^2)**					
**Mean (SD)**	27.5 (5.68)	28.7 (5.98)	28.1 (5.86)	27.8 (8.22)	26.9 (6.88)
**Median [range]**	27.1 [17.0,49.2]	27.8 [18.2,52.6]	27.4 [17.0-52.6]	26.5 [14.8, 51.9]	26.4 [14.2, 53.1]
**Waist Circumference (cm)**					
**Mean (SD)**	91.2 (14.3)	99.2 (16.0)	95.6 (15.7)	90.9 (18.1)	92.5 (15.4)
**Median [range]**	90.1 [59.4, 133]	96.6 [47.4, 157]	93.8 [47.4-157]	87.3 [64.4, 148]	90.4 [60.5, 157]

BMI, body mass index; NHOPI, Native Hawaii or other Pacific Islanders
